# Bone density of first and second segments of normal and dysmorphic sacra

**DOI:** 10.1186/s10195-020-00545-9

**Published:** 2020-05-25

**Authors:** Joseph M. Radley, Brian W. Hill, Daemeon A. Nicolaou, Stephen B. Huebner, Kelby B. Napier, Dane H. Salazar

**Affiliations:** 1 Department of Orthopaedic Surgery, University of Pittsburgh Medical Center-Hamot, 201 State Street, Erie, PA 16550 USA; 2 Department of Orthopaedic Surgery, Saint Louis University Medical School, 3635 Vista Ave, Saint Louis, MO 63110 USA; 3 Department of Radiology, Saint Louis University Medical School, 3635 Vista Ave, Saint Louis, MO 63110 USA; 4 Department of Radiology, Mallinckrodt Institute of Radiology, Washington University, 510 S Kingshighway Blvd, Saint Louis, MO 63110 USA; 5 Department of Orthopaedics, Loyola University, Chicago, 2160 S 1st Ave, Maywood, IL 60153 USA

**Keywords:** Iliosacral screws, Pelvic fracture fixation, Pelvic ring disruptions, Sacral dysmorphism, Regional bone density

## Abstract

**Background:**

Iliosacral screw fixation is safe and effective but can be complicated by loss of fixation, particularly in patients with osteopenic bone. Sacral morphology dictates where iliosacral screws may be placed when stabilizing pelvic ring injuries. In dysmorphic sacra, the safe osseous corridor of the upper sacral segment (S1) is smaller and lacks a transsacral corridor, increasing the need for fixation in the second sacral segment (S2). Previous evidence suggests that S2 is less dense than S1. The aim of this cross-sectional study is to further evaluate bone mineral density (BMD) of the S1 and S2 iliosacral osseous pathways through morphology stratification into normal and dysmorphic sacra.

**Materials and methods:**

Pelvic computed tomography scans of 50 consecutive trauma patients, aged 18 to 50 years, from a level 1 trauma center were analyzed prospectively. Five radiographic features (upper sacral segment not recessed in the pelvis, mammillary bodies, acute alar slope, residual S1 disk, and misshapen sacral foramen) were used to identify dysmorphic characteristics, and sacra with four or five features were classified as dysmorphic. Hounsfield unit values were used to estimate the regional BMD of S1 and S2. Student’s *t*-test was utilized to compare the mean values at each segment, with statistical significance being set at *p* < 0.05. No change in clinical management occurred as a result of inclusion in this study.

**Results:**

A statistical difference in BMD was appreciated between S1 and S2 in both normal and dysmorphic sacra (*p* < 0.0001), with 28.4% lower density in S2 than S1. Further, S1 in dysmorphic sacra tended to be 4% less dense than S1 in normal sacra (*p* = 0.047). No difference in density was appreciated at S2 based on morphology.

**Conclusions:**

Our results would indicate that, based on BMD alone, fixation should be maximized in S1 prior to fixation in S2. In cases where S2 fixation is required, we recommend that transsacral fixation should be strongly considered if possible to bypass the S2 body and achieve fixation in the cortical bone of the ilium and sacrum.

**Level of evidence:**

Level III.

## Introduction

Osseous fixation pathways within the pelvis have been well described [1]. The iliosacral screw corridor has been increasingly utilized for management of pelvic ring injuries [2–8]. The technique for implantation of iliosacral screws has been shown to be safe and effective when performed properly [5–18]. When employed with closed reduction and percutaneous insertion, this technique can rapidly stabilize the pelvis with minimal morbidity for the patient [2–8]. However, placement of iliosacral screws requires a detailed understanding of sacral anatomy. Previous anatomic studies revealed an anatomic variation that differs from the “normal” phenotype. This “dysmorphic” variant has anatomic restraints that limit iliosacral fixation into the first sacral segment (S1), while being more open to fixation in the second sacral segment (S2) [15–18].

While screw insertion into both S1 and S2 has been shown to be safe, little has been written on the bone density of each sacral segment. Computed tomography (CT) scan has attracted interest as a means of evaluating bone mineral density (BMD) from studies ordered for other diagnostic purposes. Recent studies have evaluated the application of this technique to the sacrum [19, 20]. Zou et al. compared CT-acquired Hounsfield unit (HU) values at S1 with validated gold-standard DEXA and CT at L1, establishing guidelines for the use of HU in the sacrum as a marker for osteoporosis [19]. Salazar et al. [20] studied otherwise healthy trauma patients showing relative osteopenia of S2 in comparison with S1, which may have implications for iliosacral screw fixation of pelvic ring injuries.

Iliosacral screw fixation failure has been described in literature associated with osteopenic bone [6, 21]. The purpose of this study is to expand upon the understanding of regional BMD of the sacrum in otherwise healthy trauma patients through stratification of sacra based upon morphology. Using CT-scan HU values, we reexamined the difference in bone density at S1 compared with S2 in both normal and dysmorphic sacra. Further, we examine whether there is a difference in density between normal and dysmorphic sacra at each level. Based upon clinical observations, we hypothesize that, in normal and dysmorphic sacra, there will be a relative osteopenia of S2 compared with S1. We also hypothesize that lower density will be found in the dysmorphic sacra compared with the normal phenotype.

## Materials and methods

This study was approved by our institutional review board. Pelvic CT scans of 50 consecutive patients between the ages of 18 to 50 years were prospectively evaluated. Patient care was not altered as a result of this study. CT data used were collected as a routine component of trauma workup. CT scans were excluded for previous documented sacral trauma, lumbar/sacral implants, sacral fracture, neoplasm of the pelvic girdle, rheumatoid arthritis, seronegative arthropathies, osteoporosis/osteopenia, paraplegia, nonambulatory/wheelchair bound status, or signs of malnutrition. Patients were also excluded for use of bisphosphonates, steroids, or hormonal medications. Exclusion criteria for inadequate scan technique limiting density determination included motion artifact, streak artifact, beam hardening artifact, or photon deprivation in the extremely obese patient. The subjects’ age and gender were recorded.

**Fig. 1:**
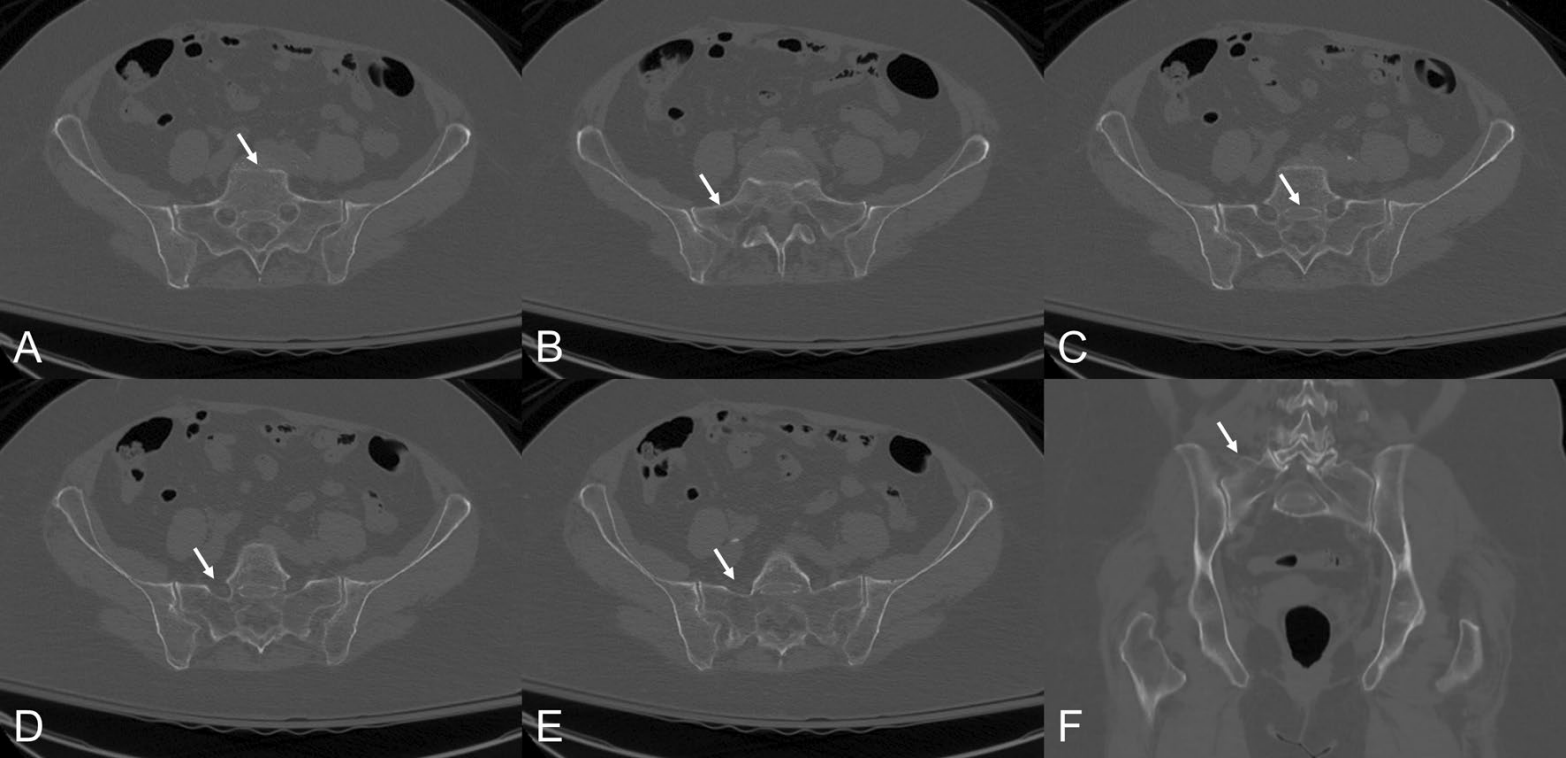
CT images showing dysmorphic features. Axial images demonstrate: (**a**) S1 not recessed in the pelvis, (**b**) acute alar slope, (**c**) residual disk, and (**d**, **e**) misshapen sacral foramina. Coronal imaging demonstrates (**f**) mammillary bodies

After identification of the patient’s CT scans, two musculoskeletal radiologists independently identified features of dysmorphic sacra. For the purposes of this study, these features included: (1) an upper segment that is not recessed in the pelvis, (2) the presence of mammillary bodies, (3) an acute alar slope, (4) a residual disc between the first and second sacral segments, and (5) noncircular upper sacral neural foramina (Fig. 1). These features were chosen based on prior study on sacral morphology with regard to sacral dysmorphism [15–18]. Sacra with four or five features were classified as dysmorphic.

**Fig. 2:**
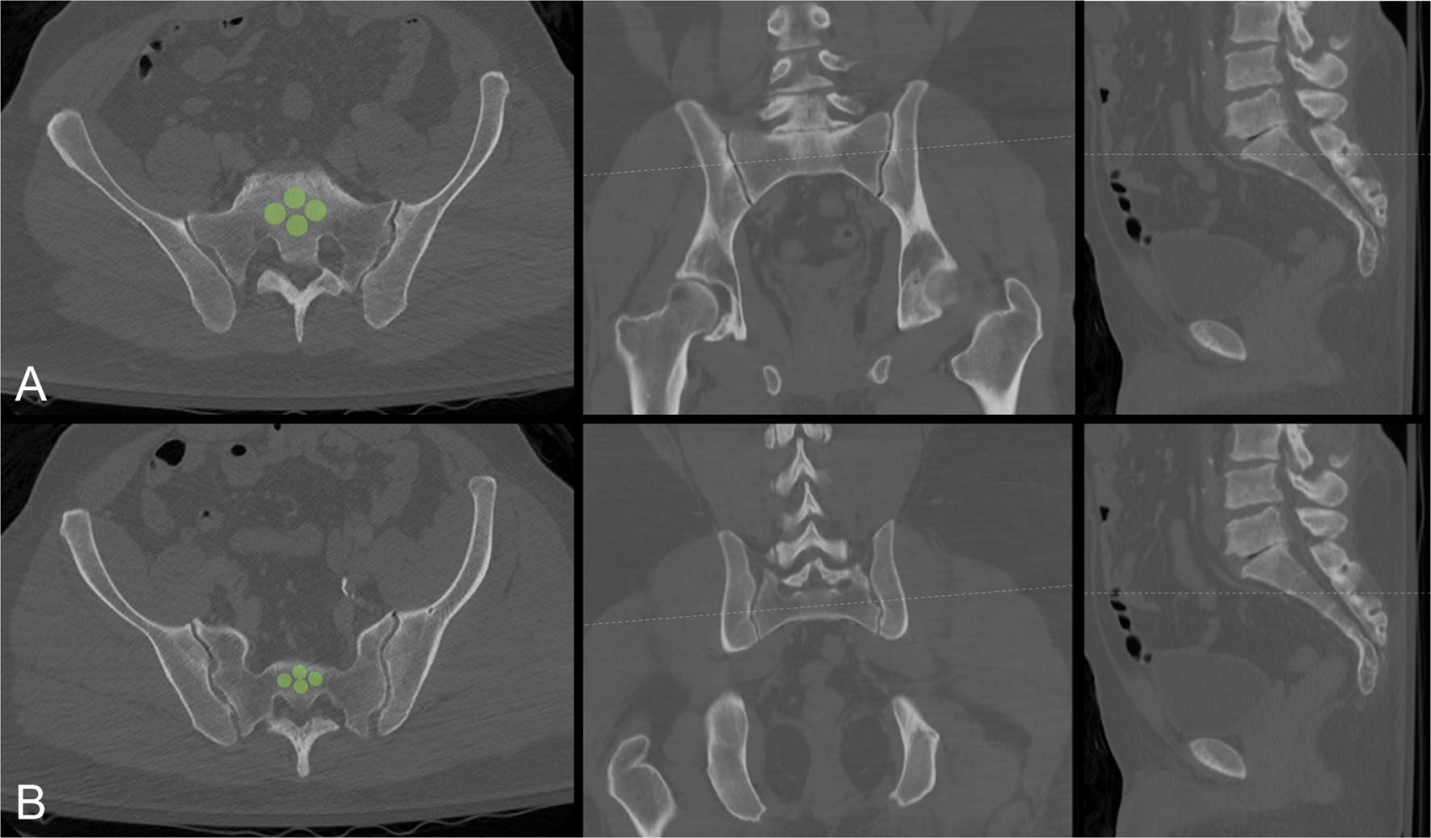
Axial, sagittal, and coronal CT images depicting cross-referencing technique used for localization of regions of interest (ROIs) represented by green circles in (**a**) S1 and (**b**) S2

A Hounsfield unit value for each sacral segment was then calculated adapting the methodology from Salazar et al. [20]. For each sacral segment, four circular regions of interest (ROIs) were strategically placed utilizing the axial CT sections (Fig. 2). The ROIs were placed into the anterior, posterior, right lateral, and left lateral aspects of each sacral body utilizing the axial CT imaging via the picture archiving and communication system (PACS) software. These were positioned to minimize overlap among the individual ROIs. Once placed, a HU value was obtained for each ROI. The four values were then averaged to arrive at a single HU value for each sacral segment.

### Statistical analysis

Prospective power analysis revealed that a sample size of 25 patients was necessary to detect a difference in S1 compared with S2 at the 0.05 alpha level with 80% power. The data collected were analyzed utilizing SPSS 25.0 (IBM Corp) statistical software. The variables were tested for normal distribution, and the data were not skewed. Student’s *t*-test was utilized to compare the mean HU values at each segment, with statistical significance being set at *p* < 0.05. Mann–Whitney *U*-test with chi-squared comparison was used to assess dysmorphic sacra. Interrater reliability was performed utilizing percent agreement and Cohen’s kappa statistics.

## Results

Two CT scans were excluded after radiographic analysis due to inadequate imaging and repeated imaging of another subject with a different electronic medical record number. The remaining 48 patients whose CT scans were analyzed had a mean age of 33.7 years (18–50 years). The majority of the subjects were male (39/48, 81%).

The mean HU for S1 (320.9 HU, 204.25–447.25 HU) was significantly greater than that for S2 (229.8 HU, 107.37–408.6 HU) (*p* < 0.0001) (Table 1). Subgroup analysis showed that this was true in both normal sacra (S1: 323 HU versus S2: 234 HU; *p* < 0.001) and dysmorphic sacra (four or more dysmorphic features; S1: 320 HU versus S2: 228 HU; *p* < 0.0001). With respect to the S1 body, the mean HU at the anterior (*p* = 0.002), right lateral (*p* < 0.001), and left lateral (*p* = 0.0017) ROIs were significantly greater than that at the posterior ROI. For the S2 body, the mean HU of the anterior ROI was significantly (*p* = 0.003) greater than those of the right lateral, left lateral, and posterior ROIs.

**Table 1:** Demographics and mean HU measures

Subjects	48
Age (years)	33.7 (18–50)
Gender	39M, 9F
Mean S1 (HU)	320.9^†^
S1 anterior (HU)	329
S1 right (HU)	333
S1 left (HU)	332
S1 posterior (HU)	287
Mean S2 (HU)	229.8^†^
S2 anterior (HU)	253
S2 right (HU)	229
S2 left (HU)	218
S2 posterior (HU)	217
≤ 3 dysmorphic features	
S1 (HU)	326^§^
S2 (HU)	230
> 3 dysmorphic features	
S1 (HU)	313^§^
S2 (HU)	225

*HU* Hounsfield units

^†^*p* < 0.0001

^§^*p* = 0.047

Further evaluation with regard to anatomical variance showed that 35/48 (72.9%) of the pelvises were identified as having at least one dysmorphic feature by the musculoskeletal radiologists (Table 2). Of the patients sampled, 5/48 (10.4%) had an upper sacral segment not recessed in the pelvis, 9/48 (18.8%) had mammillary bodies, 6/48 (12.5%) had acute alar slope, 34/48 (70.8%) had a residual disk, and 6/48 (12.5%) had misshapen sacral foramen. Thirteen (27%) were identified as having no dysmorphic features. Twenty-four (50%) were identified as having one characteristic, 3/48 (6%) as having two characteristics, 3/48 (6%) as having three characteristics, 2/48 (4%) as having four characteristics, and 3/48 (6%) as having all five characteristics. When there were four or more dysmorphic features identified, the mean HU at S1 tended to be less than that of subjects with three or fewer dysmorphic features (313 HU versus 326 HU, respectively; *p* = 0.047). There were no HU differences at the S2 body regardless of the number of dysmorphic features.

**Table 2:** Prevalence of dysmorphic sacral features

Upper sacral segment not recessed in the pelvis	10.4%
Mammillary bodies	18.8%
Acute alar slope	12.5%
Residual disk	70.8%
Misshapen sacral foramen	12.5%
Dysmorphic features	
0	27.08%
1	50.0%
2	6.25%
3	6.25%
4	4.17%
5	6.25%

The interrater reliability between the two musculoskeletal radiologists showed substantial to excellent agreement for four of the five dysmorphic features. Cohen’s kappa failed to show such agreement for the residual S1 disk (Table 3).

**Table 3:** Interrater reliability

	Agreement (%)	Kappa value
Upper sacral segment not recessed in the pelvis	71	0.810–0.911
Mammillary bodies	67	0.606–0.650
Acute alar slope	67	0.704–0.765
Residual disk	81	0.104–0.829
Misshapen sacral foramen	55	0.592–0.728

## Discussion

We confirmed our first hypothesis showing that the average density of S2 was 28.4% lower than S1. A subgroup analysis showed similar results. These findings corroborate the earlier results by Salazar et al. [20] in a study of 25 normal sacra in which S2 was 28.1% less dense. Further analysis of our results showed that density was highest in the anterior and lateral ROIs of S1. Although the ROI in the posterior aspect of S1 was found to have a lower density than the remainder of S1, this was found to have higher density than all aspects of S2.

Our second hypothesis that dysmorphic sacra would have lower BMD was confirmed in S1 but not in S2. Our study results show that S1 in dysmorphic sacra tended to have lower density than the normal morphology. The explanation for this difference is not elucidated by our dataset, but we believe that differences in anatomy may alter the biomechanics of force transmission during weight bearing and thus density based upon Wolff’s law. Further study on the biomechanics of force transmission in normal versus dysmorphic sacra would be needed to confirm this; however, while statistically significant, only a 4% decrease was found, which may lack clinical significance in regards to biomechanical impact on fixation strength. S2 showed no difference in density based on morphology, which may be a result of more anatomic similarity at S2 compared with S1.

Prior biomechanical studies on pelvic ring injuries have shown improved stability with multiple iliosacral screws [22, 23]. Our results would support that the most dense bone for fixation would be the anterior aspect of S1, followed by the posterior aspect of S1, when possible. Unfortunately, the S1 anatomy is not always amenable to multiple points of fixation, in particular in sacra with dysmorphic features. In these sacra, the S1 corridor is 36% smaller and typically lacks a safe transsacral S1 corridor. The S2 corridor is generally more open to fixation, with roughly twice the cross-sectional area. This limits iliosacral fixation options in S1 and encourages fixation into the less dense S2 [17, 18].

Sacral dysmorphism has been reported in 41–44% of the population [17, 18]. This reported prevalence is higher than in our experimental patient set (10.42%), using greater than three features for identification. To our knowledge, there is no specific criteria for designation of a sacrum as dysmorphic. Gardner et al. [17] used the overall appearance of the sacrum rather than a specific number of features for identification. Our data show a significant diversity of sacral anatomy based on identification of dysmorphic features. We believe that this highlights a need to place less emphasis on defining dysmorphism by specific radiographic features and more emphasis on the clinically relevant S1 osseous corridor anatomy.

For this study, we utilized opportunistically obtained CT scans during the initial trauma assessment. CT scan has been shown to be a powerful tool in evaluating bone density. Early studies comparing DEXA with quantitative CT scans demonstrated that CT was capable of accurately estimating regional cancellous bone mineral density [24, 25]. More recent studies have assessed bone mineral density utilizing CT scans obtained for other diagnostic reasons [19, 20, 26–29]. Zou et al. showed that HU values obtained from CT scans of the sacrum can be effectively used to assist in diagnosis of osteoporosis. Setting a cutoff value of 222 HUs at S1, this was a 90% sensitive test for the diagnosis of osteoporosis [19]. Diagnosis of osteoporosis in this study was based on DEXA and HU values at L1, which has been validated by Hoel et al. [29]. Our data would indicate that the bone present in the body of S2, averaging between 225 and 230 HUs, is only slightly more dense than the proposed 222 HUs cutoff for osteoporosis in S1. This raises concerns about the quality of bone available for fixation in S2 when fixation in S1 is limited.

There are limitations to this study. The patient population was predominantly young and male, which limits the application of these findings broadly. Despite this, iliosacral screw fixation is more commonly performed in young men. Thus, the specific study of a young male population strengthens the application of these findings to the realistic environment of trauma centers.

There are also limitations in using CT as a measure for regional bone density. While CT is able to delineate quantitative bone mineral density, this does not provide a qualitative measure of the physical cancellous microarchitecture. Invasive bone biopsy would be necessary to evaluate this.

This study reaffirms a relative osteopenia of S2 in comparison with S1, regardless of sacral morphology. The clinical significance of this difference would require biomechanical study. However, this raises concerns in particular for the management of unstable pelvic ring injury in patients with dysmorphic sacra. The limited fixation options in S1 lead to the increased need for fixation in the less-dense S2. We would argue that, in general, fixation should be maximized in S1 prior to fixation in S2, based on bone mineral density. In cases where fixation in S2 is utilized, strong consideration should be given to transsacral fixation, where fixation is achieved in the far-sided cortical bone rather than in the less-dense cancellous bone of S2.

## Data Availability

Not applicable.

## Abbreviations

S1: First sacral segment
S2: Second sacral segment
BMD: Bone mineral density
CT: Computed tomography
HU: Hounsfield unit
ROI: Region of interest
PACS: Picture archiving and communication system
DEXA: Dual-energy x-ray absorptiometry
